# Soft Tissue Schwannomas of the Hard Palate and the Mandibular Mentum

**DOI:** 10.1155/2017/7401631

**Published:** 2017-01-04

**Authors:** Cennet Neslihan Eroglu, Serap Keskin Tunc, Omer Gunhan

**Affiliations:** ^1^Oral and Maxillofacial Surgery Department, Faculty of Dentistry, Yuzuncu Yil University, Van, Turkey; ^2^Department of Pathology, Gulhane Military Medical Academy, Ankara, Turkey

## Abstract

Schwannomas are benign, slow growing, encapsulated tumours that originate from the Schwann cells. Intraoral schwannomas are rare, and most of these tumours involve the tongue. They are rarely located in the hard palate or in the facial soft tissue. Herein, we present the clinical and histological features as well as the prognoses of two male patients with schwannoma, one of which was localized to the hard palate and the other to the facial soft tissue around the mandibular mentum and caused swelling.

## 1. Introduction

Schwannomas, also called as neurilemmomas, were first reported in the early 20th century in the field of pathology. Schwannomas which arise from the Schwann cells of peripheral nerve sheaths can develop in any nerve trunk in the body as encapsulated lesions, with 25%–40% being located in the head and neck region. They primarily affect young- to middle-aged adults, especially females [1–3]. Although intraoral schwannoma is mainly located at the base of the tongue, it can be seen at the palate, buccal mucosa, gingiva, and lips [4]. Herein, we present the clinical findings, treatment, and prognoses of two cases of schwannomas encountered in the hard palate and in the facial soft tissue around the mentum of the mandible.

## 2. Case Presentation

### 2.1. Case 1

**Figure 1:**
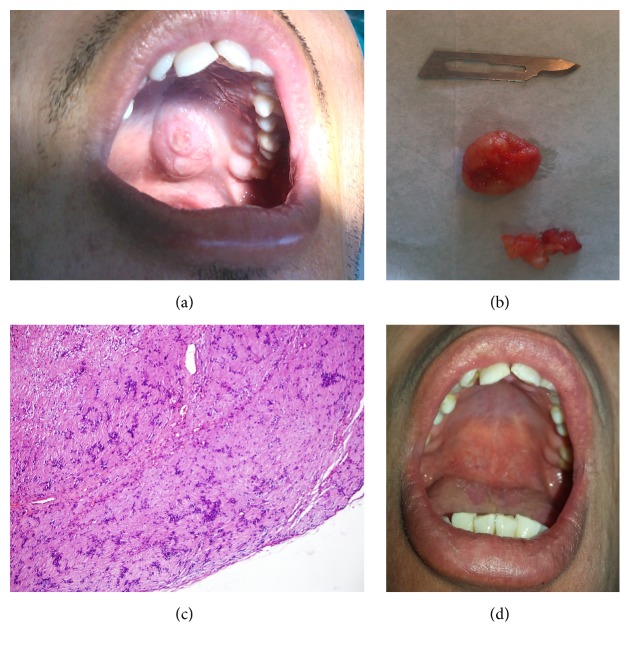
Images of the first case: (a) preoperative image of the oral cavity, (b) enucleated material, (c) schwannoma with massive cellular and fibrotic stroma (40x magnification), and (d) image of the oral cavity at the postoperative 18th month.

An intraoral mass was diagnosed incidentally in a 29-year-old male patient who was admitted to the hospital with tooth pain. The patient did not recall the time when the mass began to enlarge, since the mass caused no functional impairment. Physical examination revealed a painless, solid, asymptomatic mass, approximately 2 × 2 cm in size with a rough surface in the left palatal region (Figure 1(a)). There was no lymphadenopathy. Radiographic examination revealed no remarkable findings. The mass was enucleated under local anaesthesia with a Y-shaped incision and primary closure was performed. The encapsulated material was sent for histopathological examination (Figure 1(b)).

Examination of the histological sections demonstrated that the tumour was composed of clusters of mesenchymal-like cells with oval fusiform nuclei, which were arranged in palisading pattern. Cellular atypia or mitotic activity was not observed (Figure 1(c)). The follow-up period (18 months) was uneventful (Figure 1(d)).

### 2.2. Case 2

**Figure 2:**
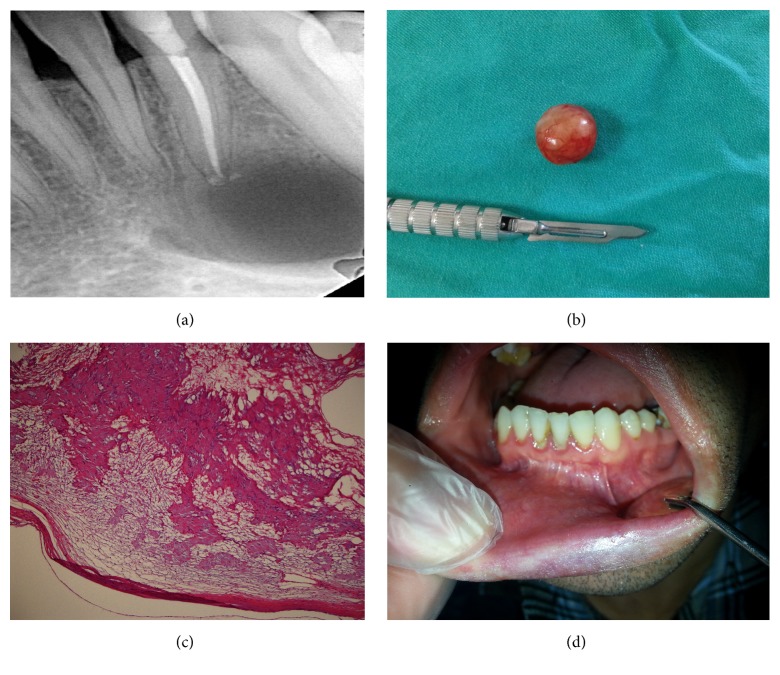
Images of the second case: (a) periapical radiographic image of the mandibular cavity that occurred secondary to schwannoma, (b) enucleated material, (c) fusiform mesenchymal cells forming cellular (Antoni type A) and loose (Antoni type B) areas (40x magnification), and (d) oral cavity at the postoperative 1st year.

A 33-year-old male patient was admitted with complaints of pain in his lower incisor and swelling at the tip of the mandible. On clinical examination, the painful, solid, and nonulcerated mass, which at first appeared to be soft tissue inflammation near the affected tooth, was observed to cause destruction in the neighbouring mandibular buccal cortex. There was no lymphadenopathy, and the patient had no systemic disease or notable medical or family history. Radiographic examination revealed a cyst-like radiolucency with a regular margin at the apex of the affected tooth (Figure 2(a)). After performing root canal treatment on the lower left lateral incisor, the encapsulated lesion was totally enucleated from the buccal soft tissue under local anaesthesia. It was observed that the lesion perforated both the buccal and lingual cortices and caused a mandibular cavity and that the lower lateral incisor was indirectly affected by the lesion and required treatment. On histological examination of the excised mass (Figure 2(b)), a collagenous lesion characterized by nonatypical cells with long, fusiform, and wavy nuclei arranged to form loose clusters was seen. There were occasional hyalinised vessels and aggregates of foamy macrophages. Cytologic atypia, high-mitotic activity, or necrosis was not observed (Figure 2(c)). The follow-up period of one year was uneventful (Figure 2(d)).

## 3. Discussion

Although it is known that schwannomas transform into tumours with the proliferation of Schwann cells, trauma is considered to be the likely unclear etiological cause [5, 6]. Intraoral schwannomas most commonly involve the tongue [7]. The currently presented cases of schwannomas are important since they were at uncommon locations. Particularly, the second case presented herein is unique and interesting in terms of its location and the type of destruction it caused. Baranović et al. [3] reported a case of schwannoma at the lingual mucosa of the mandible that caused secondary erosion of mandible. In that case, the erosion was beneath the mass, in direct contact; however, in our case, there was a healthy mucosal lining in the internal surface and at the anterior aspect of the mandible between the erosion and the lesion.

Since schwannoma is a slow-growing and painless tumour, it is very unlikely to detect schwannomas of small size. In general, patients become aware of the tumour when it reaches a size that causes functional or aesthetic problems. There may be paraesthesia in half of the schwannoma cases [8]. Although the first patient presented herein had a lesion at the hard palate, it was detected incidentally when the patient was admitted due to tooth pain. Interestingly, it caused no primary complaints, although it was in such a location that it might have caused problems in eating and swallowing. The second case was admitted with tooth pain due to the lesion and complained of facial swelling due to tooth-related abscess.

The different histopathological types of schwannomas include common, plexiform, epithelioid, cellular, and ancient schwannomas; however, mainly they have two distinct histological patterns: Antoni types A and B [1, 9]. Antoni type A areas are characterized by hypercellular proliferation of fusiform cells, while Antoni type B areas consist of a few fusiform cells in a myxoid stroma, in which not only degeneration areas, oedematous, necrotic, and haemorrhagic tissues, but also cystic formations are observed [10]. The cases presented herein were composed of Antoni type A structures without signs of degeneration.

Major pathologies, to which a number of authors recommend to pay particular attention in the differential diagnosis, include neurofibromas, traumatic neuroma, fibroma, benign salivary gland tumour, and granular cell tumour [11, 12].

Schwannomas are encapsulated tumours and, therefore, can be easily excised, and the risk of relapse is low. The rate of malignant transformation is low; however, extended dissection may be necessary in case of malignant transformation [13, 14]. In simple cases, conservative surgery performed by preserving the nerve of origin would be an adequate and appropriate treatment [15]. Although relapse is rare, the patient should be followed up for the possibility of relapse.

In conclusion, we think that schwannoma cases presented herein will contribute to the literature. Pathologies of soft tissues can lead to destructions on adjacent surfaces not only due to invasion but due to compression. Therefore, in addition to odontogenic factors, surrounding soft tissue should also be investigated by computerized tomography or magnetic resonance imaging while searching for primary tumour site in the oral cavity.
